# MYH9 Inhibition Suppresses TGF-β1-Stimulated Lung Fibroblast-to-Myofibroblast Differentiation

**DOI:** 10.3389/fphar.2020.573524

**Published:** 2021-01-13

**Authors:** Xionghua Sun, Mei Zhu, Xihua Chen, Xiaogang Jiang

**Affiliations:** Department of Pharmacology, College of Pharmaceutical Sciences, Soochow University, Suzhou, China

**Keywords:** pulmonary fibrosis, lung fibroblast, TGF– β1, MYH9, Smad2/3

## Abstract

Previous cDNA microarray results showed that MYH9 gene expression levels are increased in TGF-β1-stimulated lung fibroblast. Recently, our proteomic results revealed that the expression levels of MYH9 protein are notably upregulated in lung tissues of bleomycin-treated rats. However, whether MYH9 plays a critical role in the differentiation of fibroblast remains unclear. Herein, we demonstrated that TGF-β1 increased MYH9 expression, and siRNA-mediated knockdown of MYH9 and pharmacological inhibition of MYH9 ATPase activity remarkably repressed TGF-β1-induced lung fibroblast-to-myofibroblast differentiation. TGF-β1-stimulated MYH9 induction might be via ALK5/Smad2/3 pathway but not through noncanonical pathways, including p38 mitogen-activated kinase, and Akt pathways in lung fibroblasts. Our results showed that MYH9 inhibition suppressed TGF-β1-induced lung fibroblast-to-myofibroblast differentiation, which provides valuable information for illuminating the pathological mechanisms of lung fibroblast differentiation, and gives clues for finding new potential target for pulmonary fibrosis treatment.

## Introduction

Fibroblasts are the primary mesenchymal cells in lung tissues, and their overactivation and differentiation into myofibroblasts are crucial to fibrosis progression induced by pulmonary toxic drugs or other lesions (Siani and Tirelli, 2014; Weng et al., 2014; Darby et al., 2016), which lead to the loss of respiratory function and finally death. The differentiation of fibroblasts into myofibroblasts is featured by the increase of *α*-smooth muscle actin (α-SMA) expression and cell cytoplasmic filament formation. In addition, the activated myofibroblasts secrete abundant collagen and fibronectin-containing extracellular matrix that accumulates and is remodeled into fibroblast foci (Scotton and Chambers, 2007). These functional changes mainly contribute to fibrosis development that is regulated by canonical and noncanonical TGF-β1 signaling (Gharaee-Kermani et al., 2009).

In, 2003, Rice et al. (Rice and Leinwand, 2003) firstly reported the roles of three isoforms of skeletal muscle myosin heavy chains (IIa, IId, and embryonic) and their enzymatic activity in lung myofibroblast biology. On the other hand, class II non-muscle myosin has three isoforms, NM IIA, NM IIB, and NM IIC, which are encoded by three different genes, MYH9, MYH10, and MYH14, respectively. Southern et al. (Southern et al., 2016) thought MYH10 might be an effector of the pro-fibrotic phenotype. These indicated that elucidating the function of muscle myosin and non-muscle myosin might provide candidate targets for the regulation of myofibroblast differentiation. However, much work about these remains to be done.

Intriguingly, only the levels of MYH9 in TGFβ1-stimulated fibroblasts change in both Kapoun’s cDNA microarray data (Kapoun et al., 2006) and our previous proteomic results (Zhou et al., 2017). However, whether MYH9 really exerts an important role in lung fibroblast-to-myofibroblast differentiation remains unclear. In this work, we explored the expression and function of MYH9 protein in TGF-β1-induced lung fibroblasts and further investigated how TGF-β1 upregulated MYH9 protein expression in lung fibroblasts.

## Materials and Methods

### Reagents

Human recombinant TGF-β1 (Cat. No. 240-B-010) was purchased from R&D (Minneapolis, MN). MYH9 siRNAs (Cat. No. 129901, siRNA ID, HSS106870, HSS106871) and negative control siRNA were acquired from Invitrogen (Waltham, MA). Negative control siRNA and Smad2 and Smad3 siRNA were bought from Shanghai Genepharma Inc. Validated Smad2 siRNA sequence (Jeon et al., 2006): sense, 5′-GUC​CCA​UGA​AAA​GAC​UUA​A-3′. Validated Smad3 siRNA (Jazag et al., 2005): sense, 5′-ATG​GTG​CGA​GAA​GGC​GGT​CAA-3′. siRNAs were transfected into cells 24 h before TGF-β1 treatment by using Lipofectamine RNAiMax reagent. Blebbistatin (SF9087), SB43152 (SF7890), perifosine (SC0227), and SB203580 (S1863) were purchased from Beyotime (Haimen, China). Rabbit anti-MYH9 antibody (ab138498), rabbit anti-α-SMA antibody (ab124694), rabbit anti-fibronectin antibody (ab45688), rabbit anti-Smad2 (ab40855), and rabbit anti-Smad3 (ab40854) were bought from Abcam (Cambridge, United Kingdom). Rabbit anti-*p*-Smad2/3 antibody (8,828) were acquired from Cell signaling technology (Danvers, MA). Rat tail collagen (sc-136157) was procured from Santa Cruz (Dallas, TA). Secondary antibodies were obtained from LI-COR (Lincoln, NE).

### Cells

MRC-5 cell line (Cat. No, CCL-171; Lot No. 62559214) was procured from the American Type Culture Collection (Manassas, VA) and maintained in MEM with 10% FBS at 37°C with 5% CO_2_. The MRC-5 cells were seeded into plates, and their density was approximately 2×10^4^ cells/mL; on the following day, the medium was changed into MEM containing 0.5% FBS and cultured overnight. Then, TGF-β1 (2.5 ng/ml) co-treated with/without blebbistantin (MYH9 ATPase inhibitor), SB43152 (ALK5 inhibitor), perifosine (Akt inhibitor), and SB203580 (p38 MAPK inhibitor) was used to treat the cells. Finally, the cells were prepared for the following experiments.

### Total mRNA Isolation and Real-Time PCR Analysis

Total mRNA of MRC-5 cells was extracted using RNA extraction kit (Vazyme) and the extracted mRNA was measured by using Nanodrop spectrophotometry (Thermo-Scientific, Wilmington, DE). Total mRNA was reverse-transcribed, and cDNA was then subjected to real-time PCR analysis. The levels of MYH9 and GAPDH mRNA expression were measured by using the SYBR Green kit (Thermo). These primers’ sequences were as follows: MYH9: sense, 5ʹ- CCTCAAGGAGC- GTTACTACTCA-3ʹ; antisense, 5ʹ-CTG​TAG​GCG​GTG​TCT​GTG​AT-3ʹ, and GAPDH: sense, 5ʹ-GCT​GGC​GCT​GAG​TAC​GTC​GTG​GAG​T-3ʹ; antisense, 5ʹ-CAC​AGT​CTT-CTG​GGT​GGC​AGT​GAT​GG-3ʹ. These primers were acquired from Shanghai Sangon.

### Western Blot Analysis

Western blot analysis was conducted as previously described (Gao et al., 2013; Cui et al., 2018). In a typical procedure, the protein samples were separated by running 6% or 12% SDS-PAGE and transferred into the PVDF membrane (Merck Millipore). Then, the PVDF membrane was blocked using 5% nonfat milk for approximately 45 min, and the membrane was incubated with the indicated primary antibody overnight. On the following day, the membrane was incubated with the specific secondary antibody for approximately 1 h. Finally, the PVDF membrane was scanned and visualized by LI-COR Imaging System (Nebraska, United States).

### Cell Immunofluorescence

F-actin immunostaining analysis was carried out in accordance with a previously reported method (Cui et al., 2018; Lu et al., 2020). MRC-5 cells were induced by TGF-β1 (2.5 ng/ml) treatment with/without MYH9 siRNA for 48 h. Then, the cells were fixed and permeabilized. The permeabilized cells were incubated in 100 nM of rhodamine phalloidin for 30 min. After they were rinsed with PBS three times, the cells were incubated with DAPI working solution (final concentration: 100 nM) for 30 s. Lastly, the cells were rinsed with PBS three times, and they were visualized and captured using an Olympus X-51 microscope.

### Assay of Fibroblast-Containing Gel Contraction Capability

Fibroblast gel contraction assay was conducted in accordance with our previous report (Cui et al., 2018; Lu et al., 2020). Briefly, MRC-5 cell suspension (1×10^5^ cells/mL) was mixed with rat tail collagen solution (3 mg/ml) at a ratio of 2:1 for fibroblast-containing collagen gel assay. The mixtures were placed in plates for 30 min. The gels were then detached and continued to culture in MEM containing 10% FBS overnight. On the following day, the media were replaced with MEM containing 0.5% FBS for 24 h. Afterward, the fibroblast collagen gels were stimulated with/without 2.5 ng/ml of TGF-β1 and 10 μM of blebbistantin for 48 h. Finally, the collagen gel surface areas were determined using ImageJ software.

### Statistics

For statistical analysis, post hoc tests with one-way ANOVA with were conducted for comparisons between groups. Error bars in the figures were used to represent standard deviation.

## Results

### TGF-β1 Stimulated MYH9 mRNA and Protein Expression in MRC-5 Cells

**FIGURE 1:**
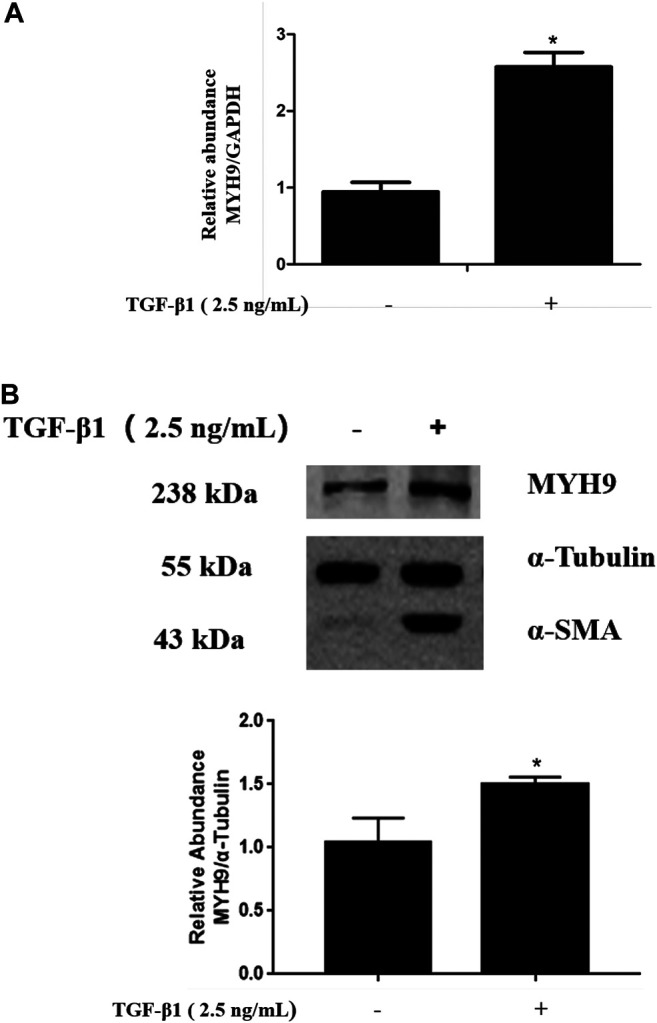
TGF-β1 stimulated MYH9 mRNA and protein expression in MRC-5 cells. MRC-5 cells were maintained in MEM with 10% FBS and stimulated with/without 2.5 ng/ml of TGF-β1 for 48 h. MYH9 mRNA expression **(A)** was determined by qRT-PCR analysis, and MYH9 protein expression **(B)** was measured with Western blot analysis. n = 3; control group vs. TGF-β1 group: *p < 0.05.

TGF-β1 is well known as a profibrotic cytokine that promotes lung fibroblast-to-myofibroblast differentiation (Kim et al., 2018). We determined the levels of MYH9 mRNA and protein expression in TGF-β1-stimulated MRC-5 cells. Both MYH9 mRNA and protein expression levels in TGF-β1-treated MRC-5 cells were higher than those of normal fibroblasts, as shown in Figure 1A and Figure 1B, which was consistent with that of previous cDNA microarray results from TGF-β1-stimulated fibroblast differentiation (Kapoun et al., 2006) and our previous proteomic results from bleomycin-treated rats (Zhou et al., 2017).

### Inhibition of MYH9 Repressed TGF-β1-Induced Fibroblast-To-Myofibroblast Differentiation in MRC-5 Cells

**FIGURE 2:**
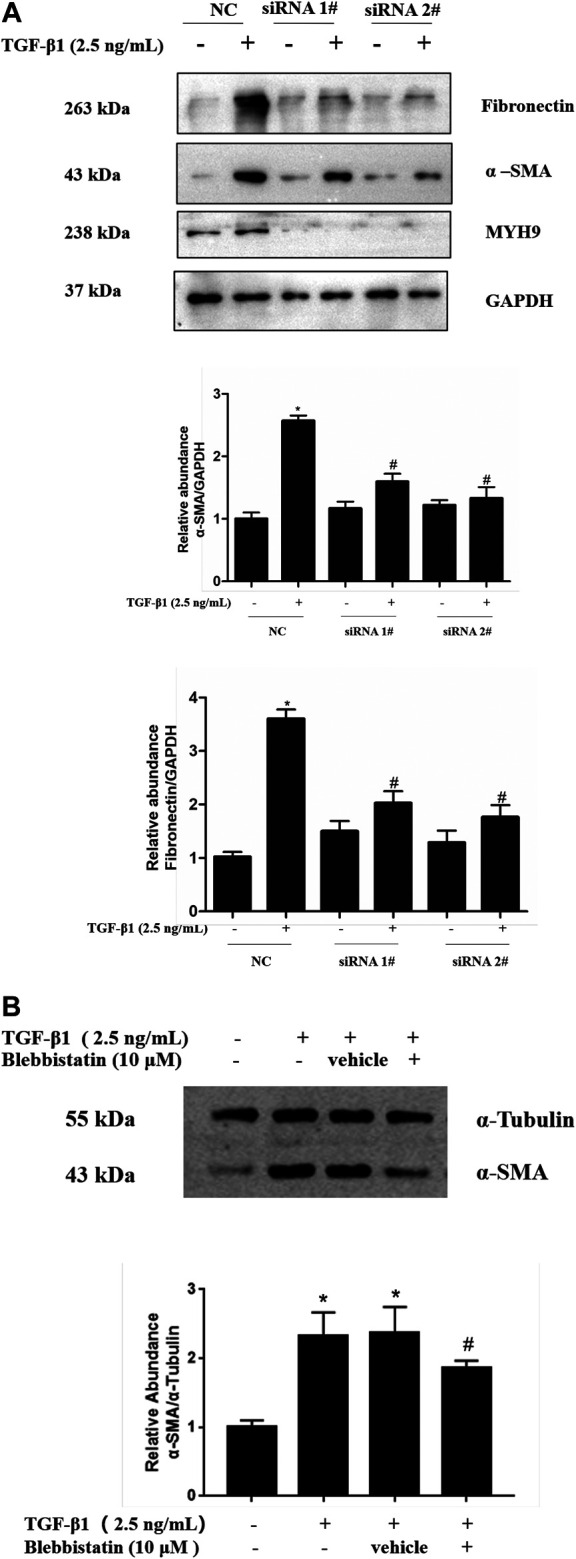
Inhibition of MYH9 repressed TGF-β1-induced fibroblast-to-myofibroblast differentiation in MRC-5 cells **(A)** RNA interference-mediated knockdown of MYH9 repressed TGF-β1-induced fibroblast-to-myofibroblast differentiation in MRC-5 cells. MRC-5 cells were transfected with scramble or MYH9 siRNA (50 nM); 24 h after transfection, the cells were starved overnight. MRC-5 cells were treated with/without 2.5 ng/ml of TGF-β1 for 48 h. MRC-5 cell protein samples were prepared and assayed by Western blot analysis for determing MYH9, *α*-SMA and fibronectin protein expression levels **(B)** Blebbistatin alleviated TGF-β1-stimulated differentiation of MRC-5 cells. MRC-5 cells were co-treated with/without 2.5 ng/ml of TGF-β1 and 10 μM of blebbistatin for 48 h. The *α*-SMA protein expression levels in different treatment groups were determined by Western blot analysis. n = 3; scramble siRNA group vs. scramble siRNA group treated with TGF-β1: *p < 0.05; scramble siRNA group treated with TGF-β1 vs. MYH9 siRNA group treated with TGF-β1: ^#^ *p* < 0.05.

To further investigate the role of MYH9 in lung fibroblast-to-myofibroblast differentiation, we conducted siRNA-mediated knockdown of MYH9 and then determined the expression levels of *α*-SMA protein, one of the most important markers of fibroblast-to-myofibroblast differentiation (Weng et al., 2014). As shown in Figure 2A, data from Western blot analysis exhibited a notable decrease in MYH9 protein upon siRNA knockdown, and subsequently MYH9 knockdown reduced the levels of *α*-SMA and fibronectin in TGF-β1-induced MRC-5 cells. This result suggested that MYH9 knockdown repressed TGF-β1-induced lung fibroblast differentiation.

MYH9 protein can integrate with actin and induce mechanical force via magnesium-dependent ATPase activity of MYH9 protein (Kovacs et al., 2004; Lv et al., 2013). Therefore, we considered whether MYH9 protein ATPase activity contributes to the TGF-β1-induced expression of fibroblast-to-myofibroblast differentiation markers in lung fibroblasts. The pharmacological inhibition of MYH9 protein by its inhibitor, blebbistatin (10 μM), prevented the induction of *α*-SMA protein by TGF-β1 in MRC-5 cells (Figure 2B). These data indicated that MYH9 inhibition alleviates TGF-β1-induced lung fibroblast-to-myofibroblast differentiation.

### Decrease in MYH9 Suppressed the Formation of Cell Filaments Stimulated by TGF-β1 in MRC-5 Cells

**FIGURE 3:**
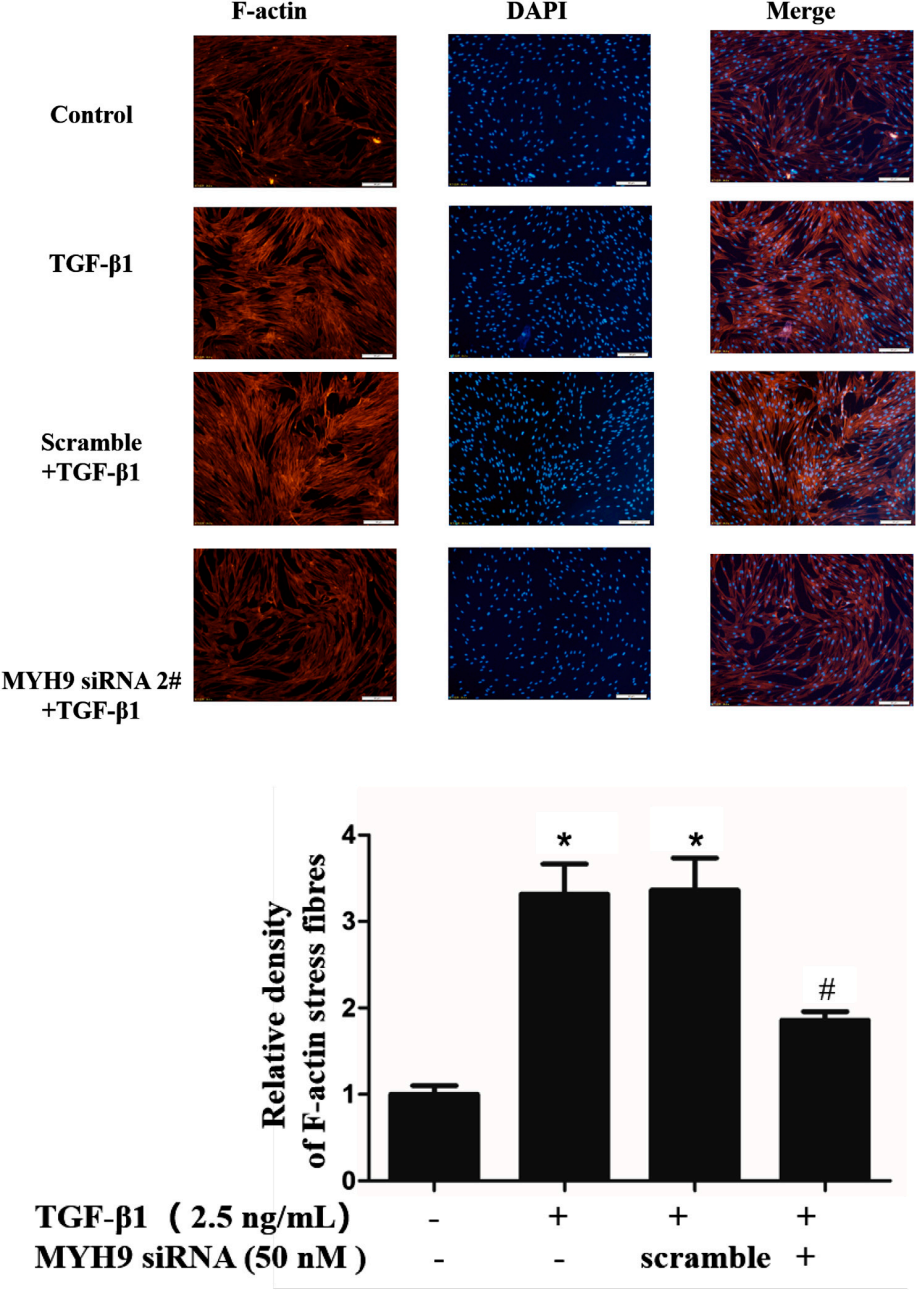
Decrease in MYH9 suppressed formation of cell filaments stimulated by TGF-β1 in MRC-5 cells. MRC-5 cells were stimulated with/without 2.5 ng/ml of TGF-β1 for 48 h. MRC-5 cells were stained with rhodamine phalloidin in red. Blue corresponds to DAPI (nuclei). Three replicate experiments were performed, and one representative experiment was displayed. The quantification results of F-actin intensity were measured using ImageJ software (n = 3, control group vs. TGF-β1 group: *p < 0.05; TGF-β1 group vs. blebbistantin group: ^#^ *p* < 0.05).

The formation of many thickened cytoplasmic stress filaments is one critical characteristic that shows a successful phenotypic transition from fibroblasts to myofibroblasts (Cai et al., 2012; Cui et al., 2018). Here, we performed MYH9 knockdown using MYH9 siRNA directed toward MYH9 mRNA to study whether MYH9 plays a crucial role in the formation of cytoplasmic filaments in MRC-5 cells. As shown in Figure 3, MYH9 downregulation abrogated the TGF-β1-stimulated well-organized cytoplasmic filaments in MRC-5 cells. This finding showed that MYH9 knockdown can repress TGF-β1-induced lung fibroblast-to-myofibroblast differentiation.

### Blebbistatin, a MYH9 Protein Inhibitor, Attenuated TGF-β1-Stimulated Fibroblast-Containing Collagen Gel Contraction Capability

**FIGURE 4:**
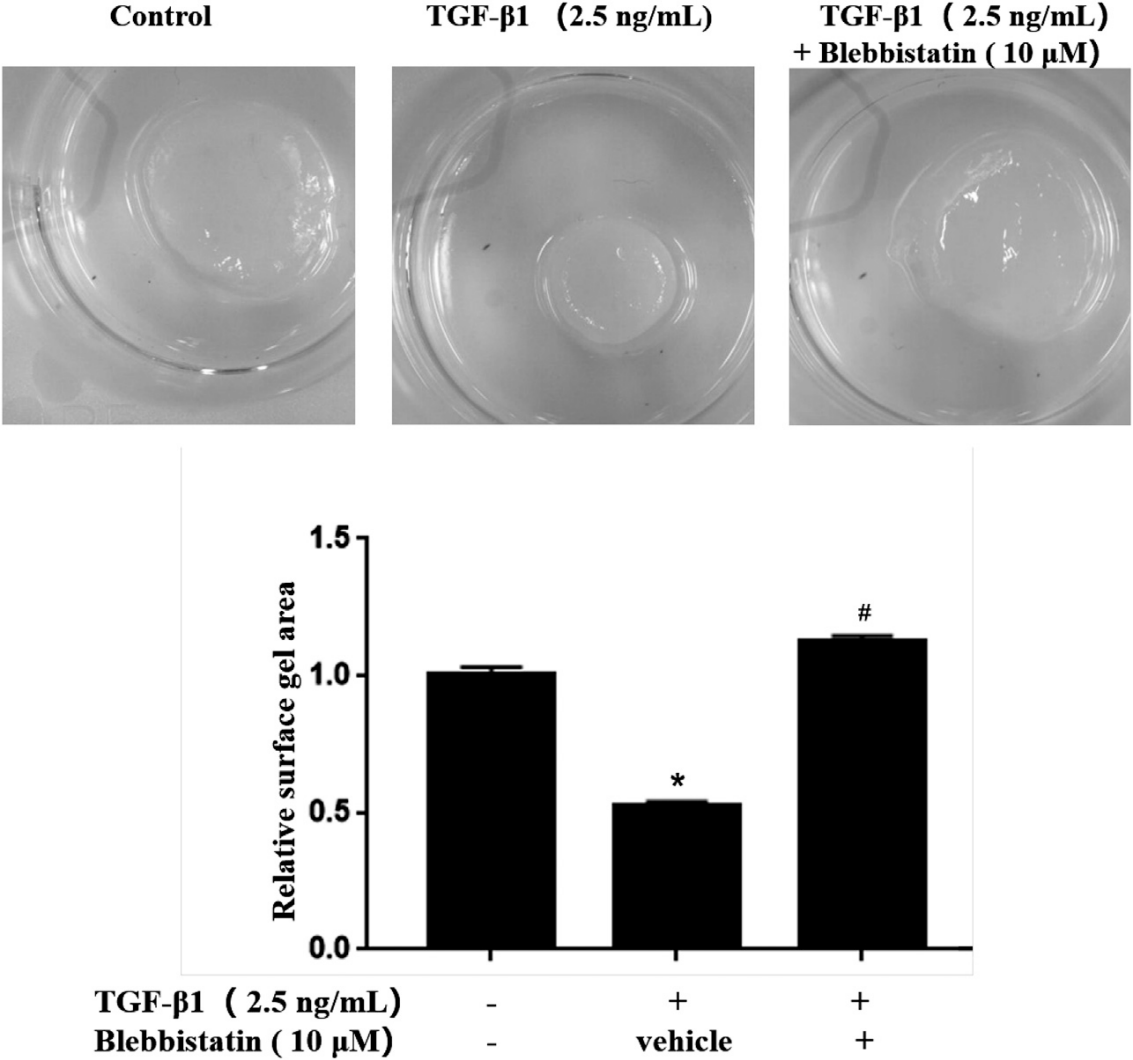
Blebbistatin attenuated TGF-β1-stimulated fibroblast-containing collagen gel contraction capability. MRC-5 cell-containging collagen gels were treated with/without 2.5 ng/ml of TGF-β1 and 10 μM of blebbistatin for 48 h. The surface area of MRC-5 cell-containing collagen gel was measured using ImageJ software (n = 3, control group vs. TGF-β1 group: *p < 0.05; TGF-β1 group vs. blebbistantin group: ^#^ *p* < 0.05).

To further validate the roles of MYH9 ATPase activity in TGF-β1-induced lung fibroblast-to-myofibroblast differentiation, we conducted collagen gel contraction capability assay as described in our previous report. As shown in Figure 4, blebbistatin decreased the contraction capacity of MRC-5 cell-containing collagen gels. The result also suggested that MYH9 inhibition attenuates TGF-β1-induced human lung fibroblast-to-myofibroblast differentiation.

### ALK5 Kinase Activity but Not the Activation of p38 MAPK and Akt Was Required for TGF-β1-Stimulated MYH9 Expression

**FIGURE 5:**
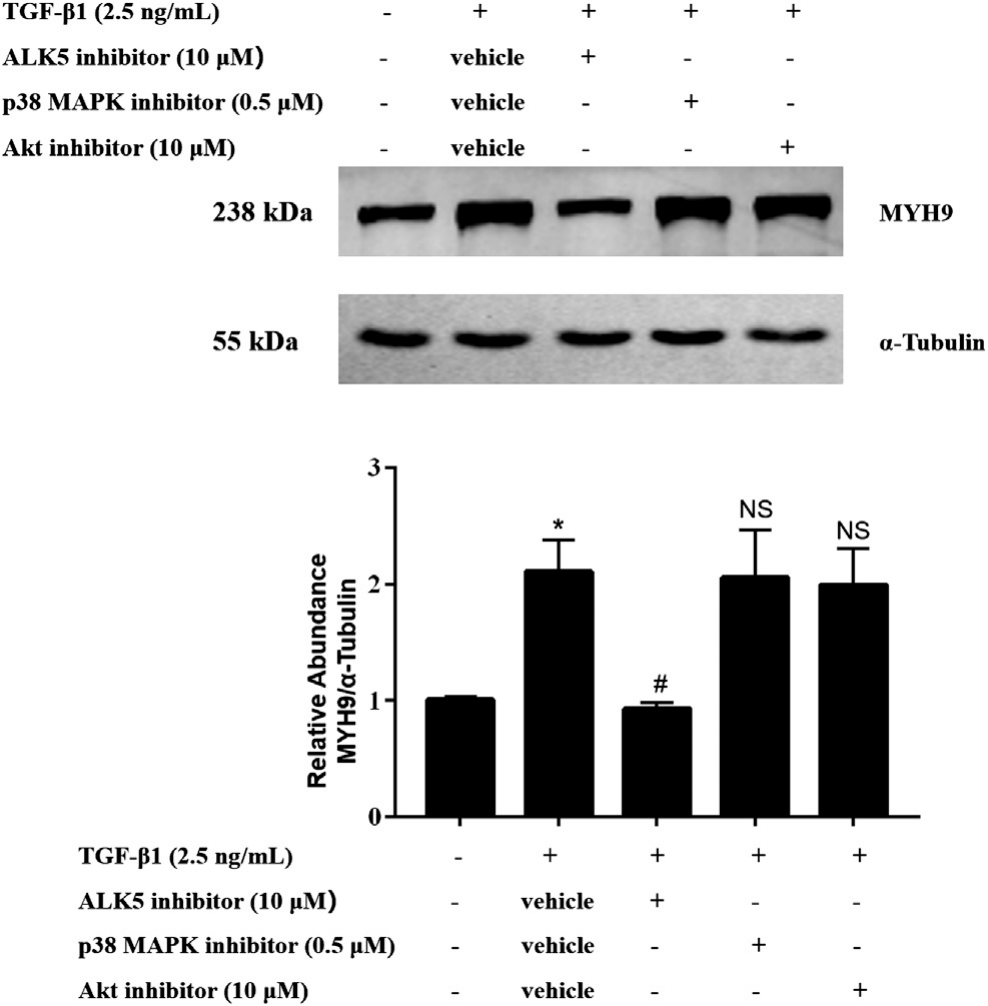
ALK5 activity but not the activation of p38 MAPK and Akt was required for TGF-β1-stimulated MYH9 induction. MRC-5 cells were co-treated with/without 2.5 ng/ml of TGF-β1, 10 μM of SB43152 (ALK5 inhibitor), 10 μM of perifosine (AKT inhibitor), and 0.5 μM of SB203580 (p38 MAPK inhibitor) for 48 h. The expression levels of MYH9 protein in different treatment groups were measured by Western blot analysis (n = 3, control group vs. TGF-β1 group: *p < 0.05; TGF-β1 group vs. ALK5 inhibitor group: ^#^ *p* < 0.05; TGF-β1 group vs. p38 MAPK or Akt inhibitor group: NS).

To define the upstream mechanisms of TGF-β1-stimulated MYH9 induction in MRC-5 cells, we tested the effects of pharmacologic inhibitors of ALK5, p38 MAPK, and Akt on these cells. Among them, only ALK5 inhibition reduced the MYH9 protein expression levels stimulated by TGF-β1 in MRC-5 cells, whereas the other two inhibitors did not affect the MYH9 protein expression (Figure 5). This result suggested that TGF-β1-induced MYH9 induction may be through ALK5 signaling activation.

### Smad2/3 Mediated TGF-β1-Stimulated MYH9 Induction in MRC-5 Cells

**FIGURE 6:**
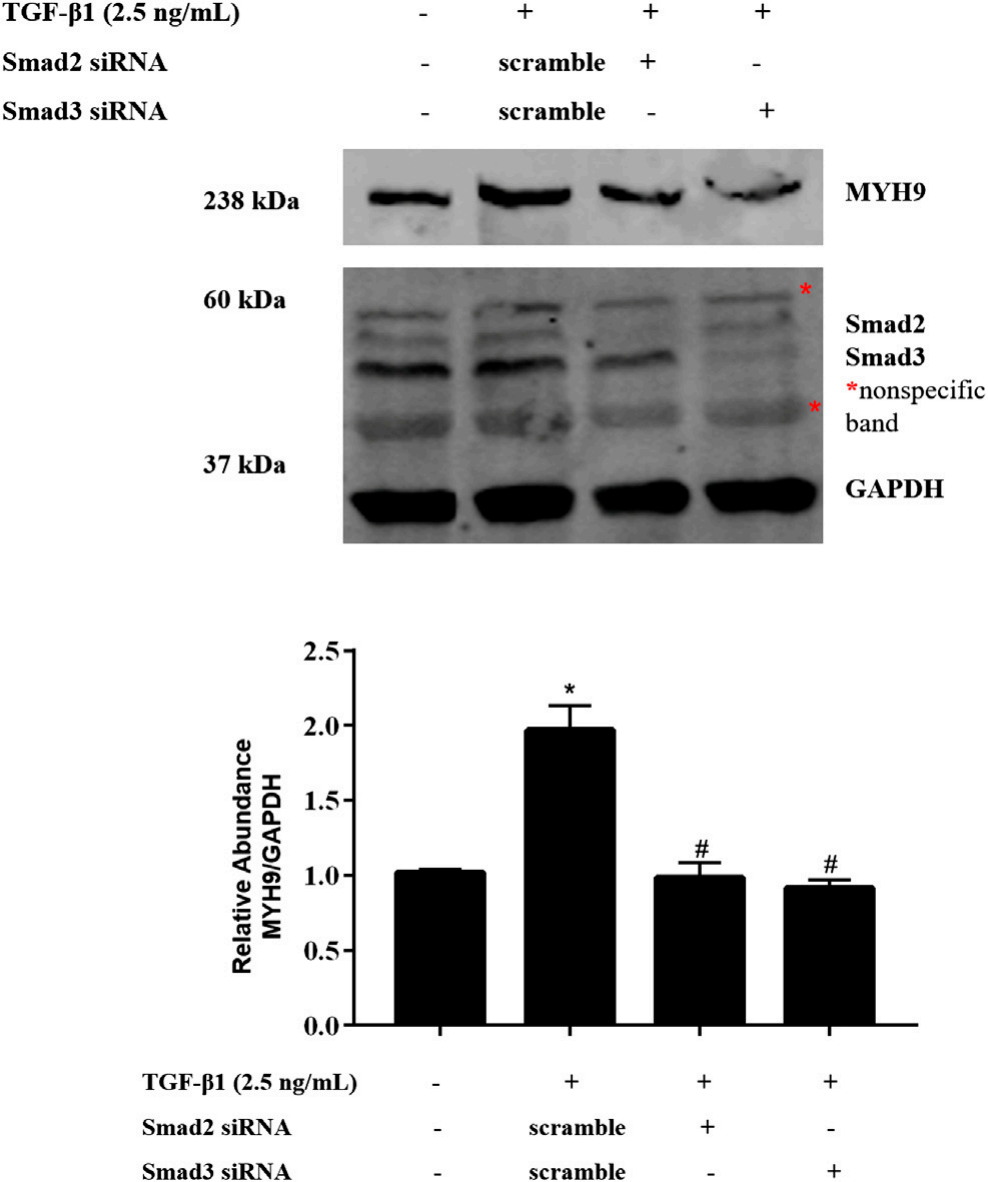
Smad2/3 mediated TGF-β1-stimulated MYH9 expression in MRC-5 cells. MRC-5 cells were transfected with scramble, Smad2, or Smad3 siRNA (100 nM); 24 h later, the cells were starved overnight. The cells were treated with/without 2.5 ng/ml of TGF-β1 for 48 h. MRC-5 cell protein samples were prepared and assayed by Western blot analysis for determing MYH9 protein expression levels (n = 3; control group vs. TGF-β1 group: *p < 0.05; scramble siRNA group vs. Smad siRNA group: ^#^ *p* < 0.05).

ALK5 is known to activate Smad2 and Smad3 (Budi et al., 2017). To determine whether TGF-β1 stimulates MYH9 via Smad2 and Smad3 protein, we used an RNAi strategy to determine if Smad2 or Smad3 is required for MYH9 induction in TGF-β1-treated MRC-5 cells; Smad2 or Smad3 knockdown inhibited TGF-β1-induced MYH9 inducibility (Figure 6). These data indicated that TGF-β1-stimulated MYH9 induction was associated with the regulation of ALK5/SMAD2/3 signaling in lung fibroblast-to-myofibroblast differentiation.

### Knockdown of MYH9 did Not Alter the Smad2/3 Phosphorylation in TGF-β1-Treated MRC-5 Cells

**FIGURE 7:**
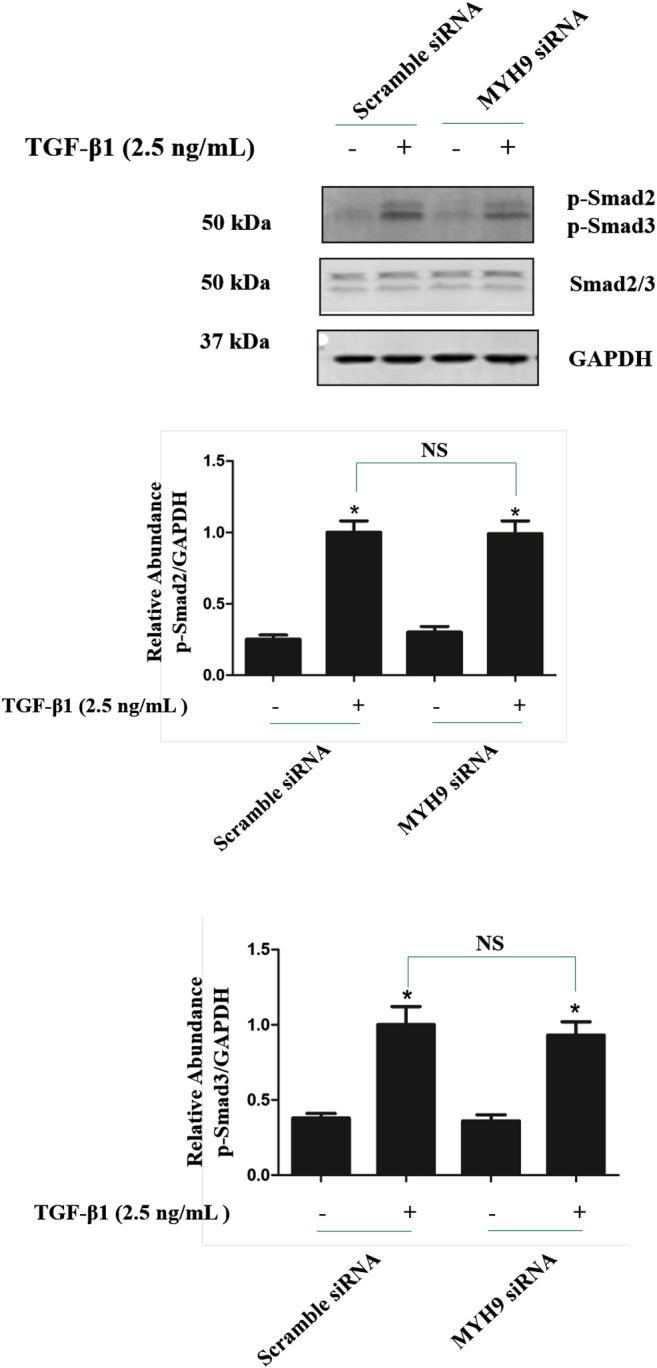
Knockdown of MYH9 did not change the Smad2/3 phosphorylation in TGF-β1-stimulated MRC-5 cells. MRC-5 cells were transfected with scramble or MYH9 siRNA (50 nM); 24 h after transfection, the cells were starved overnight. MRC-5 cells were treated with/without 2.5 ng/ml of TGF-β1 for 1 h. MRC-5 cell protein samples were prepared and assayed by Western blot analysis for determing the levels of Smad2/3 phosphorylation (n = 3, control group vs. TGF-β1 group: *p < 0.05; Scramble siRNA group vs. MYH9 siRNA group: NS).

To investigate whether MYH9 regulates TGF-β1 signaling, the levels of Smad2/3 phosphorylation were measured by immunoblotting after siRNA-mediated knockdown of MYH9 followed by TGF-β1 induction for 1 h. As shown in Figure 7, knockdown of MYH9 did not changed the phosphorylation of Smad2 and Smad3 in TGF-β1-stimulated MRC-5 cells. These data suggested that MYH9 was a downstream effector of TGF-β/Smads signaling in lung fibroblast-to myofibroblast differentiation.

## Discussion

The activation of fibroblasts is an important pathophysiological mechanism in pulmonary fibrosis (Park et al., 2012; Rahaman et al., 2014; Wei et al., 2017; Penke et al., 2018). Myofibroblasts are active effector cells in wound repair and healing, but the overactivation of these effector cells in injured or damaged tissues can dysregulate repair and healing process featured by the promotion of progressive tissue remodeling and fibrosis (Tanaka et al., 2010; Xie et al., 2016). Fibroblast-to-myofibroblast differentiation is mainly mediated by TGF-β1, but its molecular mechanisms remains unclear (Kim et al., 2018). Understanding the mechanisms is crucial for the discovery of novel therapeutic treatments for this fatal and largely treatment-ineffective disorder (Dunkern et al., 2007; Michalik et al., 2013; Sabatini et al., 2013; Yang et al., 2014; Abdalla et al., 2015). Kapoun et al. used global gene expression data for the classification and characterization of pulmonary fibrosis-specific gene sets that regulate fibroblast processes involved in fibrotic pathogenesis and found that the MYH gene is upregulated by TGF-β1 in human lung fibroblasts (Kapoun et al., 2006). In this study, we demonstrate for the first time that MYH9 is increased and is required for the induction of fibroblast-to-myofibroblast differentiation by TGF-β1 in human lung fibroblasts.

MYH9 protein, a cytoplasmic nonmuscle myosin, plays important roles in human development and disease (Pecci et al., 2018). Although MYH9 functions have been studied for decades, its role in lung fibroblast-to-myofibroblast differentiation remains elusive. In this work, we found that MYH9 inhibition represses TGF-β1-stimulated lung fibroblast-to-myofibroblast differentiation. MYH9 knockdown by siRNA led to a decrease in *α*-SMA protein expression levels in the presence of TGF-β1 and reduced TGF-β1-stimulated cell cytoplasmic filament formation in MRC-5 cells. The biological activity of MYH9 also depends on its capacity to bind and hydrolyze ATP, which drives the closed conformation of the MYH9 chaperone that represents its active form. Studies have demonstrated that the inhibition of MYH9 activity remarkably impairs cell migration and invasion. Liu et al. reported that blebbistatin, an inhibitor of MYH9 ATPase activity, decreases contraction capability of activated hepatic stellate cells (Liu et al., 2010). In our work, blebbistatin reduced the *α*-SMA expression levels in TGF-β1-treated MRC-5 cells and alleviated the contraction capability of fibroblast-containing collagen gel that was stimulated by TGF-β1. Overall, these data indicate that MYH9 inhibition can suppress lung fibroblast-to-myofibroblast differentiation. Although TGF-β1-treated MRC-5 cell is a classic model of studying fibroblast differentiation, it is not identical with fibroblasts from idiopathic pulmonary fibrosis patients, and much work about MYH9-regulated fibroblast differentiation remains to be investigated.

In the present work, MYH9 was confirmed as the protein upregulated by TGF-β1 stimulation, which is consistent with our previous proteomic data in bleomycin-treated rats (Zhou et al., 2017). TGF-β1 is a multifunctional cytokine that plays a major role in the pathogenesis of pulmonary fibrosis (Kim et al., 2018). TGF-β1 sends signals via two heterodimeric transmembrane receptors, namely, type II and type I (ALK5) receptors. TGFBR2 activates TGFBR1 (ALK5), which then induces Smad2 and 3 phosphorylation in what is known as the canonical pathway of TGF-β1 signaling. TGF-β1 can also stimulate noncanonical signaling pathways, such as p38 MAPK signaling and PI3K–Akt, which are also known as Smad-independent pathways. Canonical and noncanonical signaling pathways have been investigated in myofibroblast activation and fibrosis (Kim et al., 2018). Here, we have demonstrated for the first time that ALK5 is involved in TGF-β1-mediated upregulation of MYH9 expression in human lung fibroblasts, whereas the pharmacological inhibition of p38 MAPK or Akt signaling has low influence. Therefore, canonical TGF-β/Smad signaling pathways may mediate MYH9 induction in TGF-β1-induced lung fibroblast-to-myofibroblast differentiation. Smad2 and Smad3 are downstream effectors of ALK5, which interact with each other and their amino acid sequences have ∼90% homology similarity, and they might also play different roles in the development of tissue fibrosis and the production of ECM (Meng et al., 2010). In this work, we showed that TGF-β1-induced MYH9 induction in MRC-5 cells may depend on both Smad2 and Smad3.

**FIGURE 8:**
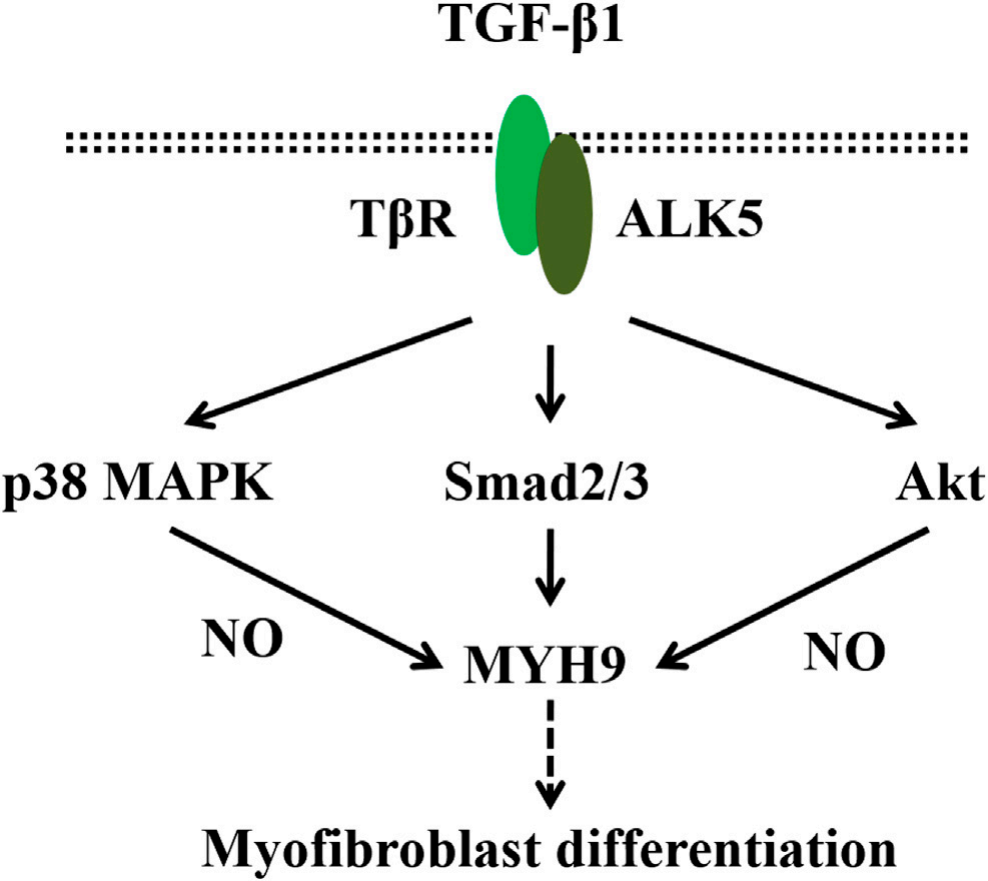
Schematic representing the regulation of MYH9 on TGF-β1-induced myofibroblast differentiation.

In conclusion, as shown in the proposed schematic representing (Figure 8), we have found that TGF-β1 can increase MYH9 protein expression, which may be primarily mediated via ALK5/Smad2/3 signaling. Furthermore, both siRNA knockdown and pharmacological inhibition of MYH9 can suppress lung fibroblast-to-myofibroblast differentiation. Further studies about the functions of MYH9 on lung fibroblast-to-myofibroblast differentiation may provide insights into the discovery of new treatment for pulmonary fibrosis.

## Data Availability Statement

The raw data supporting the conclusions of this article will be made available by the authors, without undue reservation, to any qualified researcher.

## Author Contributions

XJ designed the study; XS and MZ performed the research; XS, MZ and XC analyzed the data; XS and XJ wrote and revised the paper.

## Funding

This work was supported by grants from National Natural Science Foundation of China (81503132, 81573483) and Priority Academic Program Development of Jiangsu Higher Education Institutions (PAPD).

## Conflict of Interest

The authors declare that the research was conducted in the absence of any commercial or financial relationships that could be construed as a potential conflict of interest.
